# Diphtheria

**Published:** 1899-10-28

**Authors:** P. Watson Williams

**Affiliations:** Physician to Clifton College; and Physician in Charge of the Department for Diseases of the Throat, Bristol Royal Infirmary.


### Hospital Clinics and Medical Progress.

### DIPHTHERIA.

By P. Watson Williams, M.D.Lond., Physician to Clifton College; and Physician in Charge of the Department for Diseases of the Throat, Bristol Royal Infirmary.

(*Continued from page 44.*)

**Post-Diphtherial Paralysis** is liable to occur at a very early date, but may be delayed in its appearance two or three months. The paralysis is generally peripheral, but in rare cases cerebral hemiplegia has occurred.

The muscles involved are chiefly those of the soft palate, of the eyeball, of the lower extremities, and of the heart. Miller[30] has recorded 494 cases, observed by him at the South-Eastern Hospital, during the years 1896-1897, and of these 185 were primary paralysis of the palate, 197 strabismus, 10 paralysis of other muscles, and 102 cases of cardiac paralysis, of which only 11 did not end fatally. The bulk of the palatal paralysis occurred between the fifth and the fifteenth day, none before the fourth. Of the oculo-motor paralysis the bulk occurred between the fourth and seventeenth days, none before the fourth; of the primary paralysis of other parts half of them occurred between the tenth and fourteenth days, and none before the tenth day. A case of primary paralysis of the palate occurred as late as the sixty-fifth day; of primary oculo-motor paralysis on the ninety-first day. The bulk of the cardiac paralysis occurred between the fifth and tenth days, a few cases occurred even as early as the second day, whilst this condition occurred in a fatal form in two cases on the fifty-fourth day, and in one case it occurred with subsequent recovery on the fifty-ninth day.

Paralysis of the limbs generally affects the legs before the arms, the latter often escaping altogether, the paralysis causing dragging of a leg or weakness of an arm, but rarely amounting to inability to use the limbs. Laryngeal paralysis may occur, and then affects the abductors sooner than the adductors of the cords. Anæsthesia of the larynx may be associated with the motor paralysis, and may lead to the escape of fluids and food particles into the trachea, owing to the absence of a laryngeal cough reflex.

As regards post-diphtheritic hemiplegias, Brannan[31] reports the one and only case known among the thousands of patients treated at the Willard Park Hospital during the previous few years, and he states that in the other 35 cases on record the lesion was thought to be hæmorrhage in seven cases and embolism in 14. In six autopsies there was embolism of the Sylvian artery in five, and hæmorrhage in the lenticular nucleus in one. He found that post-diphtheritic cerebral hemiplegia is most common in children from eight to 15 years of age.

**Course.**—In cases which do not succumb the membrane ceases to form after a variable interval, and it then soon disappears, being either shed in large or small shreds, or, becoming more diffluent, is lost in the secretions of the fauces. The mucous membrane is left swollen and red, but soon becomes normal. In severe cases the swelling of the fauces and palate may be very great indeed, and this may take some days to subside; in others, superficial or even deep sloughing ulceration of the tonsils or fauces, or perforation of the palate may occur. Relapses are rare, but sometimes arise before the patient recovers completely, a fresh membrane being deposited after the disappearance of that first formed, with recurrence of the usual symptoms; and though a relapse is seldom so severe as a first attack, it may be followed by fatal results.

A recurrent sore throat with the formation of false membrane may occur some months after recovery. Gee[32] states that a recurrent pellicular angina is very prone to occur, and the recurrences may be frequent; for example, once every two or three months for a year or two, or even as many as four attacks in twelve weeks. He observes that the appearance of the throat is that of slight diphtheria (or follicular tonsillitis); but the precise nature of the disease is doubtful; this recurrent pellicular angina is very often a very febrile disorder, is not attended by albuminuria, nor followed by paralysis.

**Chronic Diphtheria.**—In very rare instances it would appear that a chronic exudate may follow an acute attack, constituting a chronic faucial diphtheria, analogous to chronic diphtheria in the nose. An instance is recorded by Jensen[33] of a nineteen-year-old servant-girl becoming ill with general symptoms and an ulcer on the right half of the soft palate, in the secretion of which virulent Loeffler's bacilli were found. During the next five months there continued to be an exudate in the pharynx in which virulent diphtheria bacilli could always be demonstrated. The bacilli were characteristically influenced by the Behring serum, while it had no effect on the exudate. The best local results were given by a simple gargle of salt water. The blood-serum of the patient protected twenty times more than normal serum against injections of Loeffler's bacilli. The persistence of Klebs-Loeffler bacilli in the fauces is sometimes long-continued, and it may cause considerable inconvenience, for though the bacilli are generally attenuated in virulence the patient cannot be pronounced safe to mix with healthy individuals so long as the specific bacillus is present and is virulent to guinea-pigs. As a result of numerous repeat examinations Hewlett and Nolan[34] find that the bacilli are commonly to be found in the throat two to three weeks after the attack—sometimes, however, as long as seven, nine, and, in one case, twenty-three weeks after convalescence; and in the latter case their virulence, as tested by inoculation of the guinea-pig, was maintained to the last. Another notable case reported by Hewlett and Nolan was that of a school-boy who six months previously had suffered from an illness which was evidently diphtheritic, and from whose throat they obtained virulent Klebs-Loeffler bacilli; "in all probability this case was the focus of infection from which originated a small outbreak of diphtheria among his class associates." Macgregor[35] found virulent bacilli in a boy's throat nearly six months after an attack of diphtheria, and Schäfer[36] records an instance where seven and a half months after an attack virulent bacilli were present. Bigg states that out of 405 cases of true diphtheria the bacilli disappeared within three days after complete separation of false membrane in 245 cases, not until seven days in 103 cases, 12 days in 34 cases, 15 days in 16 cases, three weeks in four cases, and five weeks in three cases. Golay[37] records an instance of apparent persistence of the Loeffler bacillus for 362 days in a boy aged five and a half years. The boy suffered from diphtheria in March, and up to August virulent bacilli were present in the throat. In September, in October, and in the following February he had relapses with virulent bacilli in the throat, and after that the parents would not consent to further examination as the child kept well. The importance of keeping the patient under observation until the complete disappearance of the Klebs-Loeffler bacilli is obvious, and when we recall the statements of Hewlett and Knight that they were sometimes able to convert the non-virulent pseudo-diphtheria bacillus into a virulent Klebs-Loeffler bacillus, the clinical import of Bigg's observations cannot be lightly set aside.

**The Prognosis** depends on so many factors that it is very difficult to make definite statements on the point. Undoubtedly the age of the patient is an important element, the affection being very fatal in children under five years of age, and up to the age of 15 the mortality remains high. The rate of mortality is highest during the first five years of life, especially between the ages of 2 and 5; the period between 5 and 10 comes next, and then that between 10 and 15.

Another very important point is the duration of the disease before the patient comes under proper treatment. The statistics of the results of antitoxin injections show conclusively that with each day that has elapsed the mortality rate ascends, and, though in less degree, the same was observed in pre-antitoxin days. Sporadic cases are less often of a malignant type than those occurring in early days of a severe epidemic, and during the course of an epidemic there is a tendency for the virulence of the cases to decline.

Rapid extension of the membranes, when associated with marked fœtor of the breath, dark grey colour of the membrane, intense swelling of the glands and tissues of the neck, extension of the membrane to the larynx or nasal passages, obstinate vomiting, cardiac weakness, and a depressed temperature are all signs of bad import; but extensive membranous formation in the fauces is not *per se* of much prognostic value.

Paralysis, except cardiac paralysis and post-diphtheritic hemiplegia, are not usually dangerous, and there is no proportion between the severity of the primary diphtheritic attack and their occurrence, and recovery from the paralysis is generally complete.

**Treatment.**—General treatment should be directed (*a*) to prevent the inoculation of other individuals by careful isolation, scrupulous cleanliness, and disinfection of all articles that may have been contaminated; (*b*) to maintain the strength of the patient by avoiding all unnecessary exertion, by the frequent administration of light, easily assimilated food, and small quantities of alcoholic stimulants; (*c*) to combat the general, and especially the cardiac depression, by the internal administration of such remedies as iron, quinine, strychnine, and digitalis, and, by the early and free administration of the antitoxin, to inhibit the action of the diphtheria toxin in the tissues. And local treatment is necessary: (*d*) to prevent the extension of the membrane and to destroy the vitality of the specific bacilli, so as to prevent formation and absorption of the poison; (*e*) to overcome the obstruction to respiration when the membrane has extended to the larynx.

**Treatment. General.**—The main indications are complete rest, the maintenance of the patient's strength with plenty of light nourishing food, the administration of drugs which counteract the tendency to cardiac failure and other symptoms as they arise, and above all to inhibit the vitality of the specific bacillus and the action of its toxins on the tissues by the free and early administration of antitoxic serum.

The patient should be confined to bed in a warm, well-ventilated apartment, however mild may be the onset of the disease, so as to give as complete rest to the heart as possible. Iron, preferably in the form of the acid tincture of the perchloride, will tend to counteract the anæmia. When the heart begins to fail digitalis and strychnia should be given, and, if necessary, with no stinting hand; but small doses, when sufficient for the purpose, are preferable to an early and undue freedom in the exhibition of these drugs, and the same may be said of alcoholic stimulants.

[30] Med. Supplement to the Report for 1897 of the Statistical Committee of the Med. Asyl. Bd., 1898, p. 185. [31] New York Med. Rec., July 30, 1898. [32] Allbutt's System. [33] Centr. f. Inner Med., 1897, No. 19; Amer. Jour. Med. Sci., Aug., 1897. [34] Brit. Med. Jour., Feb. 1, 1896. [35] Lancet, Mar. 12, 1898. [36] Brit. Med. Jour., vol. i., p. 65, 1895. [37] Rev. Med. de la Suisse Rom., Nov. 20, 1897; Brit. Med. Jour., Jan. 22, 1898.

